# English Speakers’ Implicit Gender Concepts Influence Their Processing of French Grammatical Gender: Evidence for Semantically Mediated Cross-Linguistic Influence

**DOI:** 10.3389/fpsyg.2021.740920

**Published:** 2021-10-15

**Authors:** Elena Nicoladis, Chris Westbury, Cassandra Foursha-Stevenson

**Affiliations:** ^1^Department of Psychology, University of British Columbia, Kelowna, BC, Canada; ^2^Department of Psychology, University of Alberta, Edmonton, AB, Canada; ^3^Department of Psychology, Mount Royal University, Calgary, AB, Canada

**Keywords:** grammatical gender, cross-linguistic influence (CLI), covert gender, lexical co-occurrence, language learning

## Abstract

Second language (L2) learners often show influence from their first language (L1) in all domains of language. This cross-linguistic influence could, in some cases, be mediated by semantics. The purpose of the present study was to test whether implicit English gender connotations affect L1 English speakers’ judgments of the L2 French gender of objects. We hypothesized that gender estimates derived from word embedding models that measure similarity of word contexts in English would affect accuracy and response time on grammatical gender (GG) decision in L2 French. L2 French learners were asked to identify the GG of French words estimated to be either congruent or incongruent with the implicit gender in English. The results showed that they were more accurate with words that were congruent with English gender connotations than words that were incongruent, suggesting that English gender connotations can influence grammatical judgments in French. Response times showed the same pattern. The results are consistent with semantics-mediated cross-linguistic influence.

## Introduction

When processing a second language (L2), learners often show influence from their first language (L1), or cross-linguistic influence (Odlin, 2005; Jarvis and Pavlenko, 2008; Serratrice, 2013; Gujord, 2020). For example, L1 speakers of languages in which complex consonants do not appear at the start of syllables (as the st- in *stupid*) sometimes produce L2 English words with an extra vowel at the beginning, like *eh-stupid* (Alkhonini and Wulf, 2018). The addition of this vowel makes the phonology of the word correspond more closely to the phonology of L1. Cross-linguistic influence (CLI) has been documented in phonology (Aoyama et al., 2004; Gildersleeve-Neumann et al., 2009; Liu, 2011; Alkhonini and Wulf, 2018), vocabulary (Sparks et al., 2009; Melby-ßLervåg and Lervåg, 2011; Yang et al., 2017), lexical choice (Navés et al., 2005; Burton, 2013; Mthethwa, 2016), morphology (Collins, 2002; Ivaska and Siitonen, 2017), syntax (Chan, 2004; Mthethwa, 2016; Ågren et al., 2021; Requena and Berry, 2021), idioms (Al-Mohizea, 2017), and even how frequently people gesture with their hands while speaking (So, 2010). CLI is not necessarily short-lived during the process of language learning: even highly fluent bilinguals show CLI (Odlin, 2005), although how exactly CLI is manifested can change with increasing proficiency in L2 (Navés et al., 2005; Gildersleeve-Neumann et al., 2009).

Cross-linguistic influence can be positive or negative (Odlin, 2005). Positive CLI means that the influence from L1 supports target-like performance in L2. For example, one recent study showed that French-English bilingual children were better able to understand passive sentences in English than would be expected given the amount of exposure to English (Nicoladis and Sajeev, 2020). The authors argued that the bilingual children’s strong performance was due to CLI from French, since passive sentences are formed identically in the two languages. Negative CLI, sometimes called interference, means that influence from L1 results in non-target-like performance in L2. For example, Nicoladis (2006) showed that French-English bilingual children were more likely to misorder adjective-noun constructions than French and English monolingual children. While English adjectives generally appear prenominally (e.g., *the big monkey* or *the purple monkey*), the default position of French adjectives is postnominal (e.g., *le singe violet*, literally “the monkey purple”). The French-English bilingual children were more likely than monolinguals to misorder adjectives in both languages, like “the monkey purple” in English than monolinguals and “le violet singe” in French (Nicoladis, 2006); in other words, they showed negative CLI.

For CLI to occur, learners must detect (not necessarily consciously) some sort of equivalence across languages (Hammarberg, 2001; Ivaska and Siitonen, 2017; Requena and Berry, 2021). In some cases, the equivalence is evident in the surface form of the languages. Cognates are words that are similar in two languages in terms of both phonology and semantics. One study found that L2 learners were more likely to show lexical CLI for cognates than for non-cognates (Burton, 2013). This result could mean that the L2 learners were sensitive to the surface-form similarities of the words. In many cases of CLI, the equivalence must be somewhat abstract. One example was already mentioned above: French-English bilingual children might be able to understand passive sentences in English at monolingual-age-appropriate levels because of the similarity of the passive construction in both languages (Nicoladis and Sajeev, 2020). While the word order for passives is identical in French and English, the words to form the passives in French and English share no surface features. To be sensitive to that similarity, children must have developed an abstract representation of a passive construction (Nicoladis and Sajeev, 2020). Another study found that children who spoke a highly inflected L1 acquired inflections in L2 English faster than children who spoke an L1 with few inflections (Blom et al., 2012). Children can only benefit from the existence of inflections in L1 when learning L2 English if they have some abstract representation of inflections. There are even some reports of CLI when there is little overlap at all in the surface form of the two languages. Some studies have found evidence for syntactic CLI across languages that do not share word order (Hatzidaki et al., 2011; Hwang et al., 2018).

What is the nature of the abstract representation across languages such that CLI occurs? One possibility, at least in some cases of CLI, is meaning (Odlin, 2005; Nicoladis, 2006). Some evidence came from a study of French-English bilingual children’s CLI in adjective-noun ordering (Nicoladis, 2006). As noted earlier, English adjectives generally appear prenominally and French adjectives postnominally. However, there are a handful of French adjectives that usually appear prenominally, like *grand* “big” and *nouveau* “new.” Nicoladis (2006) found that bilingual children were more likely to show negative CLI from French with English adjectives that usually appear postnominally in French (like *purple*) than with adjectives that appear prenominally (like *big*). Nicoladis (2006) pointed out that for this pattern of CLI to occur, bilingual children are likely relying on the semantic similarity of adjectives across their two languages. Odlin (2005) summarized other instances of CLI in adults that are based on similarities of meaning across languages.

The purpose of the present study was to test the hypothesis that L1 English semantics would influence the processing of L2 French grammatical gender (GG). GG refers to the classification of nouns into a small number of categories (often masculine, feminine, and neuter). Some languages, such as French or Greek, mark every noun for GG while other languages, like English, do not (Vigliocco et al., 2005; Bender et al., 2011). GG sometimes coincides with natural gender (the word for *cow* is often feminine across languages) but not always. In Greek, the words for daughter and son are, respectively, feminine and masculine, while the words for girl and boy are both neuter. Mark Twain (1880) famously complained that:

“In German, a young lady has no sex, while a turnip has. Think what overwrought reverence that shows for the turnip, and what callous disrespect for the girl.” (Retrieved from https://en.wikisource.org/wiki/A_Tramp_Abroad/Appendix_D)

It is not entirely clear if GG involves a semantic/conceptual consideration for native speakers of languages with GG. If so, then speakers of gendered languages might associate characteristics of natural gender with GG. Some results support that conclusion (Boroditsky et al., 2003; Vigliocco et al., 2005; Kurinski and Sera, 2011; Semenuks et al., 2017). However, in a recent review of the literature, Samuel et al. (2019) found that there was no systematic evidence for a relationship between GG and thought.

In explaining these variable results, some researchers have argued that grammatical structure may have, at most, a weak influence on thought. Instead, there may be other variables, related to culture, that have stronger influences (Braun, 1999; Mazuka and Friedman, 2000; Nicoladis and Foursha-Stevenson, 2012; Beller et al., 2015; Bender et al., 2016, 2018; Sharifian, 2017). Bender et al. (2018) found that gender connotations, beliefs about natural gender, were more powerful than GG in predicting German speakers’ responses on a Simon task based on gender associations (GA). A similar suggestion comes from Lucy (1996), who argued that discourse relativity, i.e., how a language is used, could have stronger influences on thought than the structure of a language. In support of this suggestion, one study found that Russian-English bilingual children classified more objects as masculine in Russian than in English and did not use the GG of the object’s label in Russian to classify objects (Nicoladis et al., 2016). The authors interpreted these results in terms of a strong masculine bias in the use of Russian. In other words, the children’s responses were sensitive to biases in language *use* more than language *structure*.

According to this reasoning, even in languages without GG, patterns in the language could influence adults’ associations between gender and objects. Although English has no GG, English speakers have intuitions about the gender of both abstract concepts and concrete objects. Wilkie and Bodenhausen (2012) showed that English speakers conceptualized even numbers as feminine and odd numbers as masculine (see also Wilkie and Bodenhausen, 2015). Similarly, Yan (2016) found that precise numbers were more strongly associated with masculinity than round numbers among English speakers. As for objects, Nicoladis and Foursha-Stevenson (2012) found that English speakers had intuitions about the gender of objects (e.g., stars are feminine; yoyos are masculine).

In this study, we modeled English speakers’ intuitions about the gender of objects. We quantified the degree to which words are gendered using a model of second-order lexical co-occurrence. First-order lexical co-occurrence refers to how often two words occur close together in a large corpus of text (Lund and Burgess, 1996; Landauer and Dumais, 1997). Second-order co-occurrence refers to the extent to which two words occur in similar contexts, independently of whether they occur close together. Because words that occur in similar contexts are likely to have similar meanings, second-order co-occurrence similarity is often used as a proxy for semantic relatedness. We used the skip-gram word2vec model (Mikolov et al., 2013a,b,c). This model used a neural network with (by convention) 300 hidden units to try to predict a word’s close neighbors in a large corpus of text. Each word’s vector, comprised of the 300 hidden unit weights, is a summary of that word’s context. By comparing the cosine similarity between the vectors of any two words, we can obtain a measure of the similarity of those two words in terms of their context of use, and hence (by extension) we can quantify their degree of their semantic association. For example, the words with the vectors closest to the word *kitten* in our model are *puppy, kittens, cat, pup*, and *puppies*, while the words closest to the word *cow* are *cows, pig, bovines, cattle*, and *bovine*. As these examples make clear, the models are not simply “synonym detectors” (puppies are not kittens) but rather measure broader semantic associations encoded in patterns of ordinary word use. Lexical co-occurrence has been shown to be useful in explaining many semantic phenomena, including lexical access (Hollis and Westbury, 2016), humor judgments of single words (Westbury and Hollis, 2019), and the N400 semantic context effect (Van Petten, 2014).

Co-occurrence models have an interesting feature: the vector obtained by averaging the values of many vectors from the same semantic category can serve as *a category-defining vector* (CDV). Proximity of a word vector to a well-defined CDV is a good measure of the word’s membership in that semantic category. For example, the closest ten neighbors to the vector defined by averaging the vectors of *child, infant, toddler*, and *baby* (not including those four words) are *newborn, babies, infants, toddlers, newborns, children, mother, boy, pre-schooler*, and *tot*.

In this paper we use the skip-gram model to define a gender CDV with one masculine and one feminine pole. While this vector is derived directly from patterns of language use, those linguistic patterns reflect wider cultural behavioral conventions that shape language use, as was argued by Wittgenstein (1953). In his discussion of Wittgenstein’s ideas, Bloor wrote:

“Verbalized principles, rules and values must be seen as endlessly problematic in their interpretation, and in the implications that are imputed to them. They are *the phenomena to be explained*. They are dependent, not independent variables. The independent variable is the substratum of conventional behavior that underlies meaning and implication.” (p. 137)

Although we will refer in this paper to the implicit gender assumptions that are measurable because (and to the extent that) they are encoded in *patterns of language use*, we consider those patterns to be an accessible reflection of complex cultural conventions (Wittgenstein’s “forms of life”) that are less easy to measure in a direct and quantifiable way.

We can use our gender CDV to test if the implicit gender of a word predicts English speakers’ intuitions about how masculine or feminine a word was. If these intuitions were genuinely influencing thought, then we should see evidence outside English words (Samuel et al., 2019). We therefore tested whether gender connotations, i.e., the masculinity or femininity of a word’s referent (as estimated using distance from a femininity CDV), in English predicted GG in French as a L2. Learning GG in French as a L2 is notoriously difficult (Bartning, 2000; Guillelmon and Grosjean, 2001). Bartning (2000) found that it was only very advanced learners of French who made gender agreement above chance. We predicted that French GG would be particularly difficult when there was interference from incongruent gender estimates from English.

## Materials and Methods

### Stimuli

To estimate the gender of a word’s referent in English, we used the publicly released Google News Corpus skip-gram matrix^1^. The matrix was developed from about 100 billion words of published news and contains vectors of over 3 million strings. We used it to construct two gender CDVs, each composed of the average vectors of 63 unambiguously gendered English words paired across gender (e.g., *brother/sister, he/she, prince/princess, son/daughter*, and *boy/girl*). The full set of 63 pairs, as well as male and female CDV cosine distances for 78,278 words, are available from https://osf.io/ux9he/, doi: 10.17605/OSF.IO/UX9HE.

**FIGURE 1:**
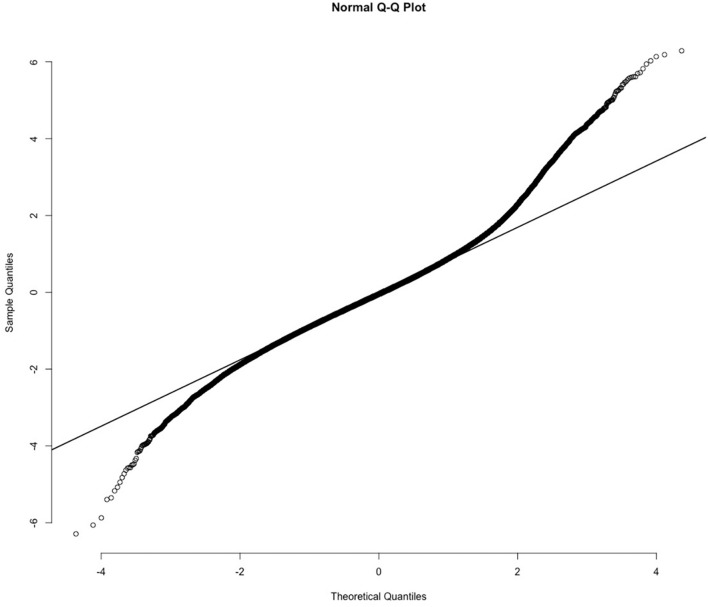
Normal expected distribution (*x*-axis) versus observed distribution (*y*-axis) of words predicted to be associated with masculinity (low negative values) or femininity (high positive values). Note: There are more words specifically associated with femininity than masculinity.

The closest neighbors of these CDVs for gender share the characteristic noted above: they do not necessarily *belong to* the semantic category of interest (i.e., masculine or feminine) but are rather *associated with* category. For example, the closest 50 neighbors to the female CDV include the masculine words *husband, boyfriend, son, siblings*, and *father*. To get a pure measure of gender, we subtracted the masculine CDV distance from the feminine CDV distance and standardized this difference. This provides us with a single standardized score for gender, with the most feminine words at one end (higher magnitude positive numbers) and the most masculine words at the other (higher magnitude negative numbers). The distribution of this measure is skewed toward femininity (see Figure 1): there are more words that are strongly gendered feminine than there are words that are strongly gendered masculine.

**FIGURE 2:**
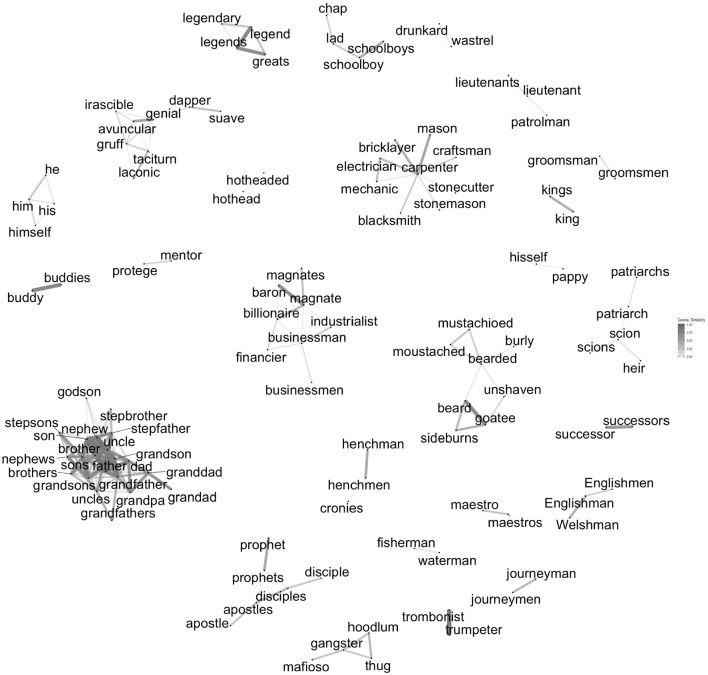
Cosine similarity between the vectors of 250 words estimated most masculine. Note: This includes all words with vectors with a cosine similarity with at least one other word’s vector > = 0.60. Words with no cosine neighbors that close are not shown. Distance between unconnected clusters is arbitrary. See also Figure 3.

**FIGURE 3:**
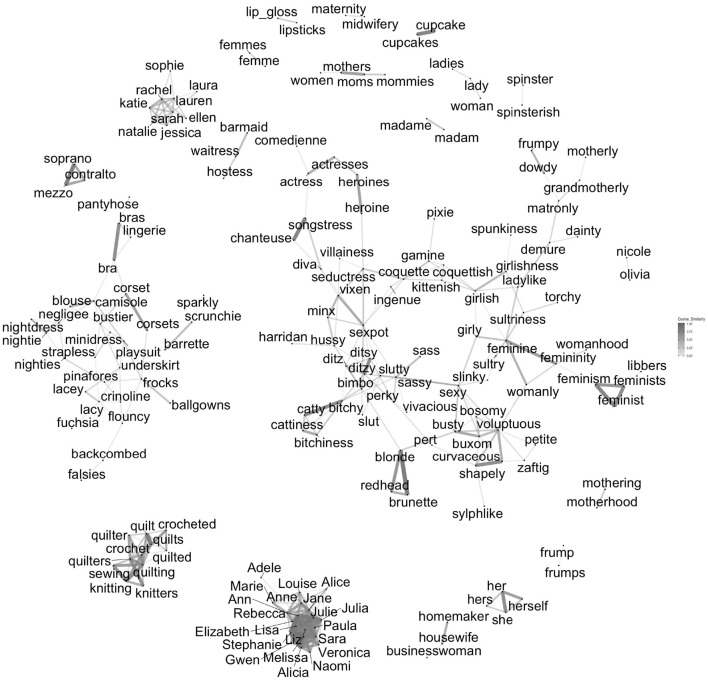
Cosine similarity between the vectors of 250 words estimated most feminine. Note: This includes all words with vectors with a cosine similarity with at least one other word’s vector > = 0.60. Words with no cosine neighbors that close are not shown. Distance between unconnected clusters is arbitrary. See also Figure 2.

The top words estimated to be most masculine and most feminine are shown graphically in Figures 2, 3, respectively. Although the figures have good face validity in our judgment, they also reveal some clear biases in language. The most feminine words include many sexual words (e.g., *lingerie, voluptuous, sexy*, and *seductress*), words associated with stereotypically feminine things (e.g., *quilting, cupcakes, lipsticks*, and *lacy*), proper names (e.g., *Adelle, Marie, Anne*, and *Alicia*), and misogynistic insults (e.g., *ditzy, slutty, bimbo*, and *bitchy*). The most masculine words are quite different in character. The largest cluster of masculine words is composed of descriptive kinship terms (e.g., *dad, nephew, stepsons*, and *uncle*). Although there are a few masculine words with negative connotations (e.g., *hoodlum, gruff, drunkard*, and *thug*), there are many with positive connotations (e.g., *legendary, mentor, magnate*, and *king*). In contrast to the feminine words, there are no masculine terms that are unambiguously sexual. The gender bias that is encoded in patterns of word use has been discussed before (Garg et al., 2018; Basta et al., 2019; Johns and Dye, 2019; Zhao et al., 2019). Since our focus is on how people are affected by the way gender is encoded in language as it is used, this bias is not problematic for our purposes. It is a reflection of the phenomenon we are studying.

**FIGURE 4:**
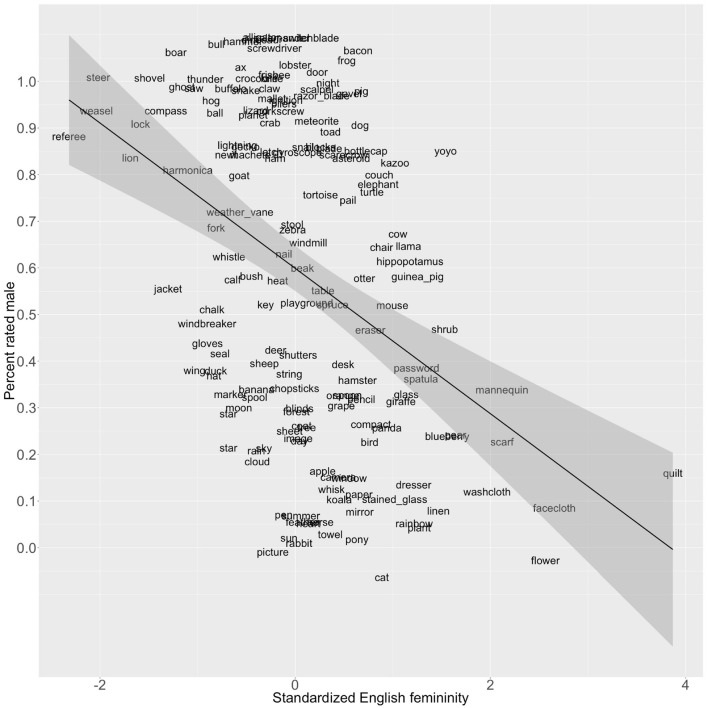
Linear relationship between the proportion of 165 common nouns judged male by 14 English speakers in Nicoladis and Foursha-Stevenson (2012) and the standardized gender estimates (*r* = -0.41, *p* = 5.43e-08). Note: Regression line shows 95% confidence intervals. Points have been randomly jittered on the *y*-axis to enhance readability.

To validate these gender estimates, we correlated them with previously obtained data from Nicoladis and Foursha-Stevenson (2012). They asked fourteen English monolinguals between the ages of 18 and 35 years to judge 174 concrete nouns according to their “first impression of whether the following objects are better classified as boys or girls” (p. 1104). Nine of those words did not appear in our dictionary. Among the remaining 165, the correlation between the proportion of times each word was classified as male and the gender estimates described above was *r* = −0.41 (95% CI: −0.53 to −0.27, *p* = 4.92e-08; see Figure 4). The correlation may be an overestimate since a large proportion of the words (52/165; 31.5%) were classified as male by at least 13/14 judges. In contrast, less than a third as many (15/165; 9.1%) were classified as female by the same criterion.

We defined words with estimated femininity more extreme than ±0.5*z* as either masculine (negative) or feminine (positive) in English. We chose sixty words that met this requirement and referred to concrete nouns. We did not include any words that were inherently masculine or feminine in English, such as *monk* or *waitress*. The sixty words were selected to fall into four categories: Masculine GG in French and Congruent with English GA (*n* = 13), Masculine GG in French and Incongruent with English GA (*n* = 15), Feminine GG in French and Congruent with English GA (*n* = 15), and Feminine GG in French and Incongruent with English GA (*n* = 17). Hereafter we refer to these categories as Masculine Congruent, Masculine Incongruent, Feminine Congruent and Feminine Incongruent, respectively. See Supplementary Appendix for list of words and gender estimates. The numbers of words were not equal across the four categories because we chose stimuli that we could later use to test Spanish learners and therefore took the GG of these words in Spanish into account.

Previous research has shown that some French words have regular gender and others irregular (Desrochers and Brabant, 1995; Taft and Meunier, 1998; Holmes and de la Bâtie, 1999; Holmes and Segui, 2004; Seigneuric et al., 2007; Lyster, 2010; Boloh and Ibernon, 2013). Regularity most often refers to how commonly words with the same ending have the same gender. For example, over 95% of words in French that end with *–ette* are feminine while 99% of words ending in *–t* are masculine (Taft and Meunier, 1998). In contrast, words ending in *–que* could be considered irregular or neutral, since both masculine and feminine words have that ending (Desrochers and Brabant, 1995). We were not able to locate data on the regularity of all of the French words included in this study (see Supplementary Appendix). Out of the 35 words for which we could find regularity, all but three are considered regular (or between easy and very easy even for French learners; see Holmes and de la Bâtie, 1999). The three exceptions were *fourmi*, *crêpe*, and *tulipe*. Participants did make less accurate gender decisions with these three words (Average correct: 61.7%) than with all the remaining words (Average correct: 74.8%; X^2^ = 24.4, *p* < 0.0001). Since they constitute such a small subset (5%) of all words in our experiment, we are not able to make other claims about differences attributable to gender regularity.

The average estimated gender for the Feminine Congruent words was 1.58*z* (SD = 0.40) and for Masculine Incongruent words 1.53*z* (SD = 0.49). The average estimated gender for the Feminine Incongruent words was −1.68*z* (SD = 0.81) and for the Masculine Congruent −1.41*z* (SD = 0.47).

**FIGURE 5:**
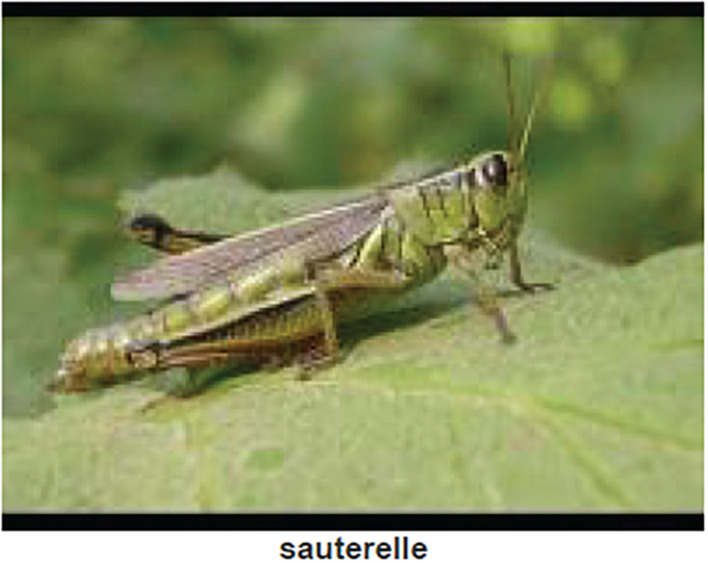
Example of an experimental item.

Each word was paired with a brightly colored photo that was sized to take up the same amount of space on the screen (see Figure 5 for an example). The target French word was placed underneath the photo. Participants could choose between *un* (the masculine singular indefinite determiner) and *une* (the feminine singular indefinite determiner). The determiners were in the same order and in the same location on the screen for all items.

### Participants

The sample included 102 undergraduate university students (84% female; 16% male; no non-binary) who had had some exposure to French as a foreign language, starting either in high school or at university. They averaged 22.3 (SD = 3.9) years of age. The participants varied considerably in their recent exposure to French, reporting from zero (*N* = 34) to 22 (*N* = 1) university courses in French. Their recent exposure was not related to their accuracy on the gender task, *r* (100) = 0.14, *ns*, so participants were included in the study despite their wide range of French exposure.

### Procedure

Students were recruited either through introductory French courses or through a student list for undergraduate students. Those interested in participating were directed to the study via a link online. The study was administered via Qualtrics. Participants first responded to two practice items (*chat* “cat,” a masculine word in French, and *lune* “moon,” a feminine word in French) so that they would get used to the layout. All participants gave the correct answer on both practice items. Response time to the first click was measured in milliseconds.

### Data Treatment and Analysis

**FIGURE 6:**
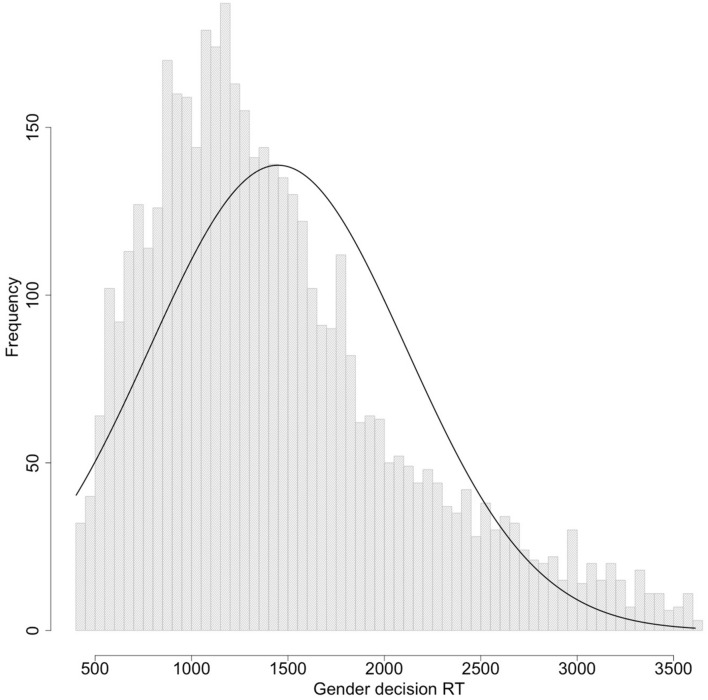
Distribution of gender decision times after data trimming.

The data include RTs as short as 19 ms and as long as 165,596 ms. Since these RTs are implausibly related to the task, we trimmed the data by removing all RTs < 400 ms or greater than 3,000 ms, and then any RTs more than 3 SDs from the average of the remaining RTs (Average [SD]: 1,479 [714] ms). In total 763 trials (13.4%) were removed for being too fast and 318 trials (5.6%) for being too slow. The remaining RTs were still long and variable, with an average of 1,448 ms and a standard deviation of 666 ms. The distribution of these RTs is shown in Figure 6.

Previous research has shown that learners of French often master agreement with masculine words before feminine words (Tucker et al., 1977; Bartning, 2000).

## Results

### Accuracy

Accuracy on the task ranged from 38.3 to 98.3% with an average [SD] of 71.6% [11.6%].

Accuracy was analyzed with generalized mixed effects modeling. Predictors or interactions were entered if they reduced the Aikake Information Criterion (Akaike, 1973, 1974) by at least five, a criterion that indicates that the model with the lower AIC was at least 12 times better at minimizing information loss than its comparator. We began with random intercepts for participants, then entered random intercepts for items. We then entered word length in English, word length in French, logged frequency in English (from Shaoul and Westbury, 2006) and logged frequency in French (from New et al., 2004). Of these predictors, only English logged frequency met the criteria for entrance into the model.

The key test for the interests of this paper is whether there is an interaction between French gender and English femininity. The hypothesis is that high English femininity should work in favor of correct decisions for French feminine words (since the English bias is in the correct direction in French) and against correct decisions for French masculine words (since the English bias is opposed to the correct response in French). We should therefore expect to see lower accuracy for French masculine words and higher accuracy for French feminine word when they are high femininity in English than when they are low femininity in English (see Table 1). The interaction was highly reliable and reduced the AIC value of the model by 30, suggesting a high probability that including it in the model reduces information loss.

**TABLE 1:** Fixed effects from generalized LME model for predicting French gender decision accuracy.

	Estimate	SE	*z*	*p*
(Intercept)	1.29	0.25	5.09	3.64E-07
English femininity	–1.24	0.39	–3.19	0.0014
Logged word frequency	0.58	0.18	3.32	0.00091
French gender [M]	–0.15	0.34	–0.46	0.65
French gender:English femininity	1.81	0.51	3.54	0.0004

*Continuous variables have been scaled. See also Figure 6.*

**FIGURE 7:**
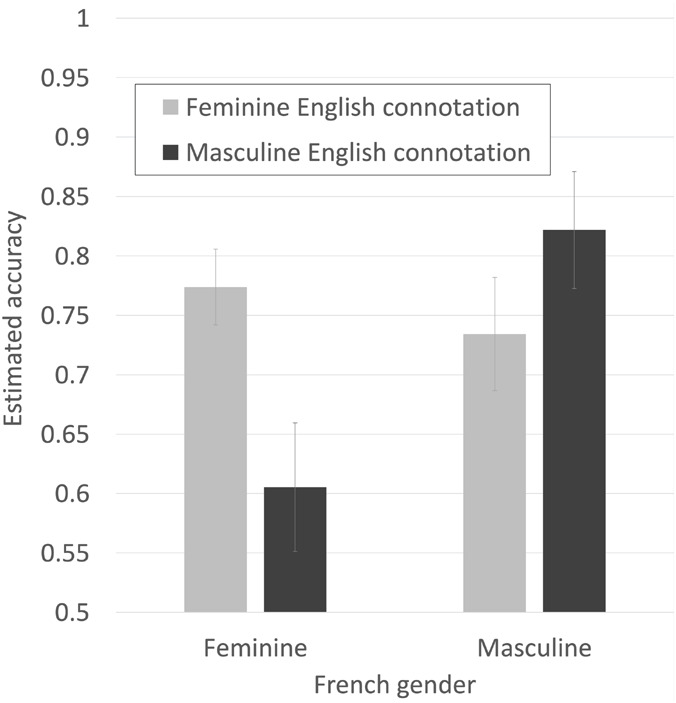
Estimated accuracy for making French gender decisions, for French feminine and masculine nouns that were either congruent or incongruent with estimated English gender connotations split high/low at their mean. Bars are SE.

As shown in Figure 7, the effects are as predicted by our hypothesis. Gender decision accuracy was higher for French feminine nouns that had feminine English connotations than for French feminine nouns that had masculine English connotations, and higher for French masculine nouns that had masculine English connotations than for French masculine nouns that had feminine English connotations.

### Response Times

We did not mention speed in the instructions to our participants. We nevertheless undertook an analogous analysis of correct decision times using linear mixed effects modeling, to test the hypothesis that congruent genders would facilitate gender judgment times. We used the same criterion of reduction of the AIC value by at least 5 for entrance into the model. Random intercepts for participant and item both entered. As with the accuracy analysis, the only fixed effect that entered the model prior to entering the key interaction was the logged frequency of the English word. Adding the interaction between French gender and English gender connotation as fixed effects decreased the AIC by 39, indicating a substantial improvement in the model’s ability to minimize information loss.

**FIGURE 8:**
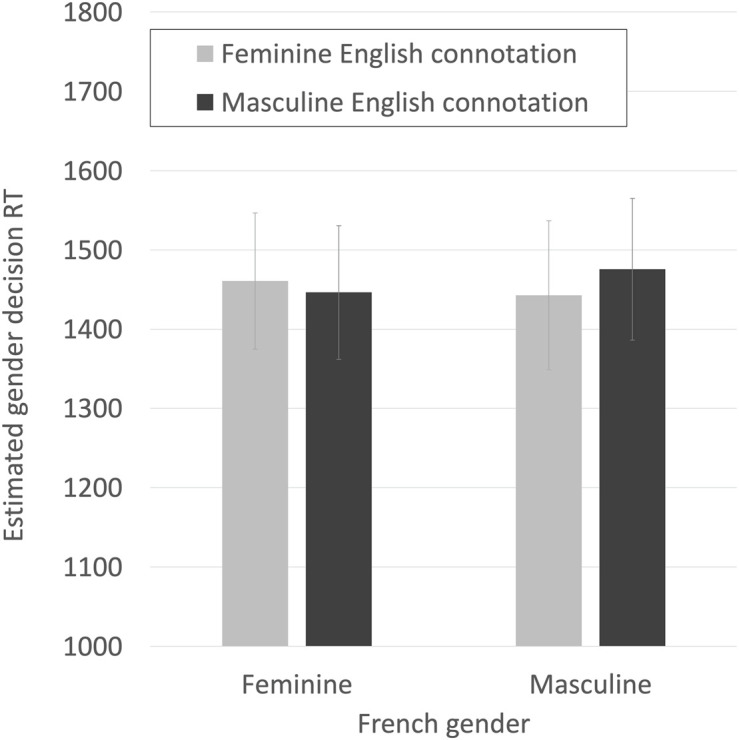
Estimated RTs for making correct French gender decisions, for French feminine and masculine nouns that were either congruent or incongruent with estimated English gender connotations split high/low at their mean. Bars are SE.

The final model for predicting RTs is shown in Table 2, with the effect shown graphically in Figure 8. The effect is small, but in the opposite direction than hypothesized. Participants were quicker (by an estimated 29 ms) to make gender decisions about French masculine words when they were high in English femininity and quicker (by an estimated 17 ms) to make gender decisions about French feminine words when they were low in femininity than when they were high. Participants were an estimated 96 ms quicker to make decisions to masculine than feminine words and showed a facilitatory effect (therefore, pulling in the opposite direction) of an estimated 106 ms per standard deviation of increasing estimated femininity.

**TABLE 2:** Fixed effects from generalized LME model for predicting French gender decision RTs for correct decisions only.

Predictor	Estimate	SE	*t*
(Intercept)	1560.12	47.2	33.05
French gender:English femininity	165.29	42.1	3.93
English femininity	–105.99	30.63	–3.46
Logged word frequency	–100.1	29.3	–3.42
French gender [M]	–96.01	42.15	–2.28

*Continuous variables have been scaled. See also Figure 7.*

**FIGURE 9:**
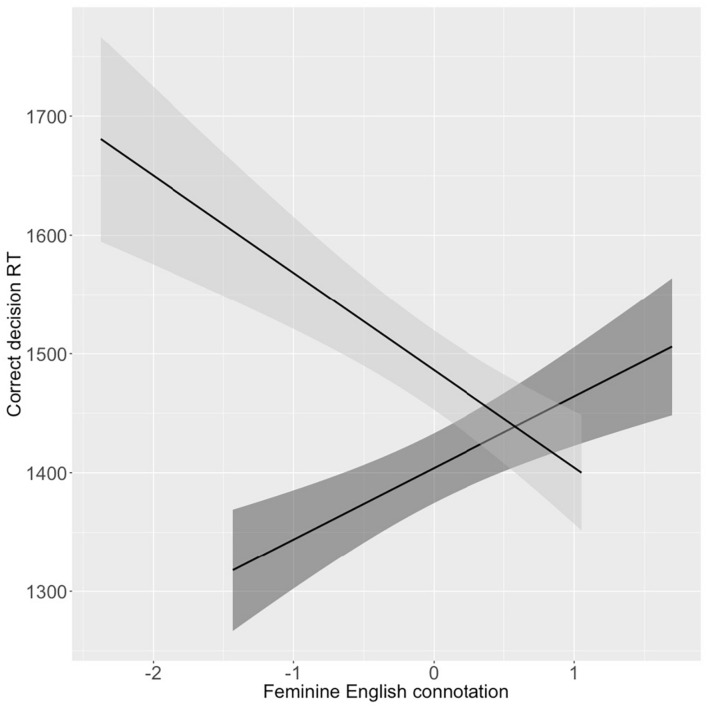
Zero-order correlations between correct gender-decision RT (*Y*-axis) and standardized estimated English feminity connotation (*X*-axis), by French gender (Dark = masculine; Light = feminine).

Figure 9 shows the zero order linear correlation of observed RT for correct gender decisions to estimated English femininity, by French GG. These zero order correlations are in the direction of the hypothesis. Words that are gendered feminine in French are correctly recognized as feminine more quickly when they are estimated more feminine in English, and words that are gendered masculine in French are correctly recognized as masculine more slowly when they are estimated more feminine in English. The figure also illustrates the problem discussed above: that RTs on this task were highly variable. This is perhaps not surprising since speed was not mentioned in the instructions.

As shown in Figure 6, this large variability in RTs is due to the fact that there is still a long tail of slow RTs after data trimming. Since gender decision is simple if you know the answer, these long RTs are likely to reflect cases in which the subject did not know the correct answer and was guessing. We re-analyzed the RT data after eliminating all RTs > 2,000 ms, leaving us with 2,829 correct responses, with as few as five and as many as 43 responses per participant. The fact that almost all responses were eliminated from some participants after eliminating both errors and very long RTs suggests that the long tail did include many participants who performed poorly at the task.

We analyzed these truncated data in the same way as we had analyzed the full dataset. Random intercepts for participant and item again entered into the model. No fixed effects (i.e., length or logged frequency in either language) entered the model prior to entering the key interaction. Adding the interaction between French gender and English gender connotation as fixed effects decreased the AIC by 33, indicating a large improvement in the model’s ability to minimize information loss.

**FIGURE 10:**
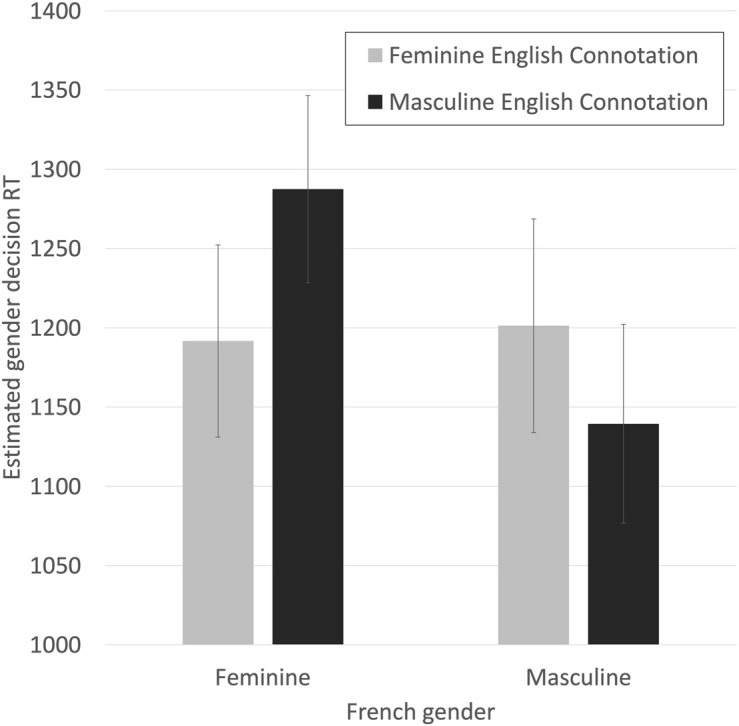
Estimated RTs for making correct French gender decisions for French feminine and masculine nouns that were either congruent or incongruent with estimated English gender connotations split high/low at their mean, using only datapoints with observed RTs < = 2,000 ms. Bars are SE.

The results, graphed in Figure 10, are as hypothesized. Correct decision times to French feminine words were estimated to be 95 ms. slower when they were inconsistent with the English gender connotation (1,287 ms.) than when they were consistent with the English gender connotation (1,192 ms.). Correct decision times to French masculine words were estimated to be 61 ms. slower when they were inconsistent with the English gender connotation (1,201 ms.) than when they were consistent with the English gender connotation (1,139 ms.). As predicted, consistency between the French gender and the English gender connotation is facilitatory in this subset of the data.

### Ruling Out Effects From French

One potential criticism of this study is that implicit gender may be encoded in French as well as English, which might nullify the key claim of influence from implicit gender reflected in L1 to GG judgment in L2. Implicit gender connotations in French are unlikely to explain our results, since most of our participants were poor French speakers, as indicated by their poor performance on the gender judgment task. Nevertheless, to rule out this possibility we undertook three further analyses.

**FIGURE 11:**
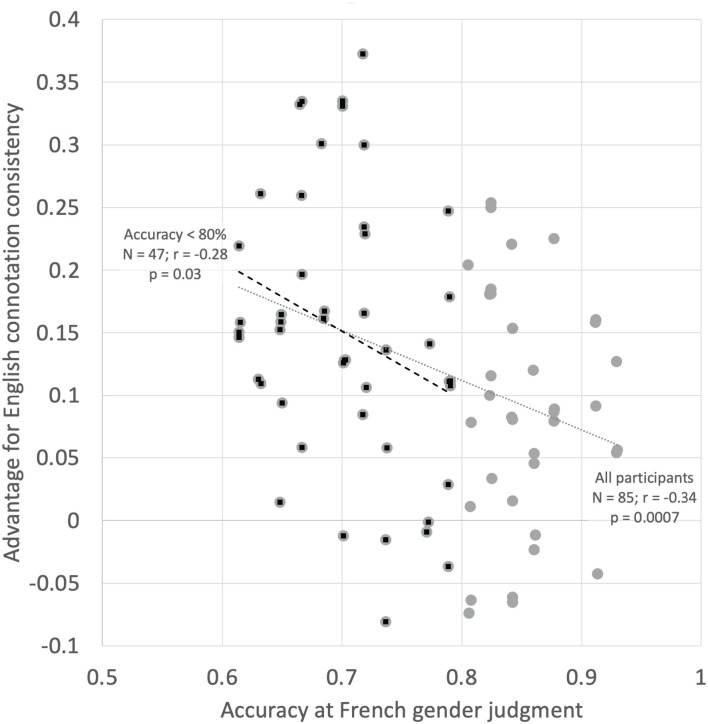
Overall performance on the French gender judgment task (*X*-axis) as a function of the size of the advantage for French/English gender congruence (*Y*-axis).

In the first analysis we examined whether the participants who performed worse at the gender judgment task (presumably, those with the least knowledge of French) were more likely to be influenced by English gender connotations. This would be expected if connotations reflected in English were driving the results, since poor French speakers have limited French semantic knowledge to draw on. To analyze this, we correlated the overall accuracy on the gender decision task with the average difference in scores between the gender-consistent word classes (French GG consistent with English gender connotation) and the gender-inconsistent word classes. The hypothesis is that there should be a negative correlation between these two measures, i.e., worse French speakers should show a stronger influence of English gender connotation (as measured by greater reliance on gender consistency) than better French speakers. The results are shown in Figure 11. There is a significant negative correlation between these measures (*r* = −0.34, one-tailed *p* = 0.0007). This result is potentially problematic since those who were highly accurate on the gender decision task necessarily have less variance by which to show an advantage for French/English gender consistency. It would be impossible to show (for example) a 30% advantage for gender consistency with an overall gender decision accuracy of 90%. We therefore repeated the analysis looking only at participants who had an average accuracy score below 80% (also shown on Figure 11). There was a significant negative correlation between the consistency advantage and overall score in this subset of participants (*N* = 47, *r* = −0.28, and one-tailed *p* = 0.03). These results are consistent with the interpretation of interference from English gender connotation to French grammatical gender.

A related analysis that does not penalize the stronger French speakers is to assess the probability of getting a perfect score in any of the gender connotation x GG categories. If English gender connotation is affecting French gender judgment, we should expect to see more perfect scores in the gender consistent cells than in the gender inconsistent cells. The relevant results are show in Table 3. A chi-square test confirms what is clear from inspection: participants were significantly more likely to get a perfect score in the gender-consistent cells than in the gender-inconsistent cells (*X*^2^ with Yates’ correction for continuity = 31.8, *p* = 8.50e-09).

**TABLE 3:** Number of participants who got a perfect score in each of the four English gender connotation × French gender cells.

	Feminine	Masculine
Female English connotation	11	0
Male English connotation	1	29

Since this measure of perfect accuracy includes both good and poor French speakers, we cannot rule out the possibility that it reflects a consistency between French gender connotation and French GG, to which strong French speakers might be sensitive. To assess this possibility, we constructed a French word2vec matrix. We built a corpus of 48.8 million words written between 2016 and 2020 by downloading and concatenating the relevant corpora from the Leipzig Corpora Collection^2^ (see Goldhahn et al., 2012). We eliminated words occurring more than 600 times per million. This notably eliminated the pronouns *il* and *elle*, which have a less straightforward semantic relationship to human gender than their English transliterations *he* and *she*, because in French these words can be used to refer to nouns other than gendered beings. We used the Gensim python library^3^ (Rehurek and Sojka, 2010) to construct a word-embedding matrix with 100 dimensions for each of 80774 words, using a neighborhood size of 2 and ignoring words that occurred less than 20 times per million. The number of dimensions is fewer than in the English matrix (300) because the corpus is much smaller. We transliterated the 63 words we had used to construct masculine and feminine CDVs in English, eliminating those that occurred more than 600 or less than 20 times per million, as well as words that translated to multiple words in French (e.g., *homme d’affaire* [businessman]; *petit fils* [grandson]). The masculine CDV was defined by averaging 50 unambiguously masculine word vectors and the feminine CDV was defined by averaging 51 unambiguously female word vectors. We subtracted the distance from masculine CDV from the distance from the feminine CDV, and normalized the resulting measure of femininity, just as we had done for the English words. We were then able to compute the standardized French-language femininity in each of the French gender × English connotation categories.

**FIGURE 12:**
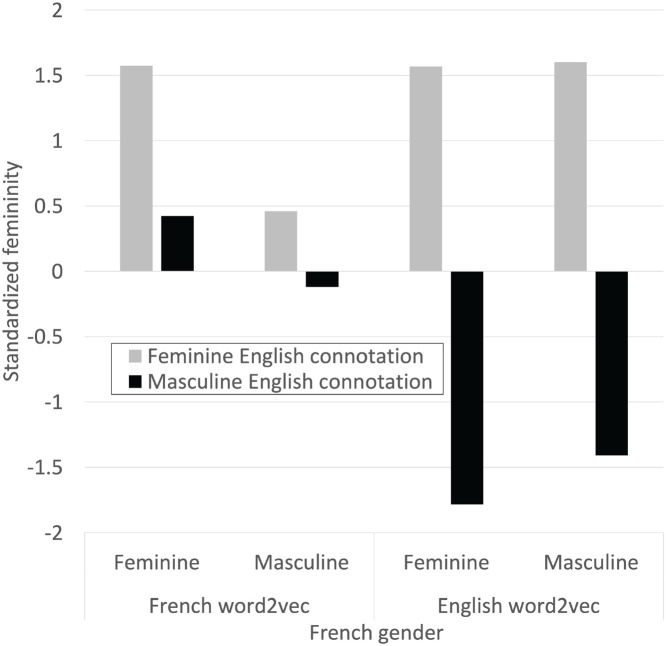
Normalized gender connotation derived from patterns of word use in French and English.

The results are shown in Figure 12, which graphs the average standardized femininity in each category in both languages. In English all four categories were deliberately constructed to contain words with standardized femininity scores with magnitudes close to or above 1.5*z*. In French, only the category of feminine words with feminine English connotations had gender connotations whose magnitude exceeded 0.5*z*. French gender connotation of the experimental words was generally weak in comparison to English and did not reflect the pattern from English. This is suggestive evidence that French gender connotation cannot account for the experimental results we have reported. It is only suggestive because it is impossible to exactly replicate the matrix from one language in another language, due to the transliteration difficulties discussed above, and because the small size of the French corpus must necessarily limit our confidence in the results.

In sum, all three of these checks on the results support our key claim, that the results on the French gender decision task do reflect gender connotations in English.

## Discussion

The purpose of the present study was to test the hypothesis that English gender connotations (resulting from associations between words) affect English speakers’ decisions about the GG of French nouns. In support of that hypothesis, we found that participants were more accurate in making decisions about French gender when that GG was congruent with the estimates of English gender connotations. This result is similar to that of Lambelet (2016), who found that the gender of the words in the L1 predicted the intuitions of gender in L2 French. However, in that study, the participants all spoke a L1 with GG. Our study demonstrates effects on GG related to a L1 that does not mark for GG.

We noted above French language learners have been shown to perform better at learning gender with masculine words than feminine words (Tucker et al., 1977; Bartning, 2000). Our data are consistent with this claim. Our participants were better at making decisions to masculine French words (77.6% correct) than to feminine French words (69.8% correct; X^2^ = 36.2, *p* < 0.0001) and were quicker in making correct decisions about masculine than feminine words [Original analyzed dataset: *t*(3163.9) = 3.6, *p* = 0.00030; Dataset with RTs < 2,000 ms: *t*(2543.9) = 4.0, *p* = 7.219e-05].

The initial RT results went in the reverse direction than hypothesized. We demonstrated that this was probably due to inclusion of participants who were very inaccurate and/or very slow. When the long tail of RTs was eliminated, the results were as hypothesized, with participants taking longer to make correct decisions to stimuli that were incongruent on the two gender measures than to stimuli that were congruent. It is important to keep in mind that we did not encourage our participants to respond quickly, so some participants may have taken time to think about their response on some items. Our RT measure was to the first response that they clicked. Although the median number of clicks was one, participants occasionally changed their answers multiple times, clicking up to four different responses. Follow-up studies could therefore emphasize to participants to respond as quickly and as accurately as possible, and perhaps use a simpler response format such as a time-limited “go/no-go” decision where participants hit one key if they believe a word’s French gender is congruent with the one they have been assigned as “go.” We predict that the RT results will mirror the results with the trimmed data in the present study.

The results are consistent with the argument that CLI can be based on shared meaning across languages (Odlin, 2005; Nicoladis, 2006). While previous studies of semantic CLI have been based on lexical semantics, we have argued that the relevant aspects of meaning in L1 in this study come from patterns of word use that reflect implicit cultural gender assumptions. The gender connotations from English patterns of use influenced French learners’ processing of GG in French.

## Conclusion

We have presented evidence for a plausible mechanism for how speakers of non-gendered languages can have intuitions about the gender of concepts and things (Braun, 1999; Wilkie and Bodenhausen, 2012, 2015; Nicoladis et al., 2016; Yan, 2016). Language users may form intuitions about the gender connotations of words based on the patterns of word use that they have encountered. Our argument is consistent with results showing that German speakers are more strongly influenced by gender connotations than by GG (Bender et al., 2018).

This study concerned only English L1 speakers. Future studies can include L1 speakers of languages with GG. If this approach proves fruitful across both gendered and non-gendered languages, it could help explain why there have been variable results in previous studies testing whether GG is related to thought (Samuel et al., 2019). In a gendered language, GG would be only one cue among many contributing to the semantic space around that word. For this reason, the choice of stimuli could have a critical impact on the results. A reanalysis of the items based on their co-occurrence may shed light on the variable results. In addition to extending these results to gendered languages, future research might extend these results to other linguistic and semantic domains, like temporal markings and pitch metaphors, in which inconsistent effects of language structure on thought have been reported (Casasanto, 2016).

Our results also have potential practical benefits for second-language learning, since they suggest an algorithmic way of identifying words whose GG may be especially difficult for English speakers to learn or remember.

## Data Availability Statement

The datasets presented in this study can be found in online repositories. The names of the repository/repositories and accession number(s) can be found below: https://osf.io/ux9he/, doi: 10.17605/OSF.IO/UX9HE.

## Ethics Statement

The studies involving human participants were reviewed and approved by Research Ethics Office, University of Alberta. The patients/participants provided their written informed consent to participate in this study.

## Author Contributions

This study was the brain child of EN and CW. CF-S designed and collected the data for the validation study. EN designed and collected the data for the French learners. CW calculated the gender estimates and ran all the analyses. EN and CW wrote the manuscript. All authors have read and approved the text.

## Conflict of Interest

The authors declare that the research was conducted in the absence of any commercial or financial relationships that could be construed as a potential conflict of interest.

## Publisher’s Note

All claims expressed in this article are solely those of the authors and do not necessarily represent those of their affiliated organizations, or those of the publisher, the editors and the reviewers. Any product that may be evaluated in this article, or claim that may be made by its manufacturer, is not guaranteed or endorsed by the publisher.

## Footnotes

1
https://code.google.com/archive/p/word2vec/

2
https://wortschatz.uni-leipzig.de/en/download

3
https://radimrehurek.com/gensim/
