# Supplementary material for: English Speakers’ Implicit Gender Concepts Influence Their Processing of French Grammatical Gender: Evidence for Semantically Mediated Cross-Linguistic Influence
Source: Front Psychol. 2021 Oct 15;12:740920. doi: 10.3389/fpsyg.2021.740920 (PMC8555711; doi:10.3389/fpsyg.2021.740920)
Supplement: Supplementary file 1 [file Table_1.docx]

Appendix. Words.

| **French word** | **English word** | **Standardized**  **English femininity** | **Standardized French Femininity** |
| --- | --- | --- | --- |
| **Feminine Congruent** | | |  |
| vaisselle† | dishes | 1.77 | 2.14 |
| poussette† | stroller | 2.11 | 0.76 |
| brosse à cheveux† | hairbrush | 1.7 |  |
| fourrure† | fur | 2.21 | 2.50 |
| couche | diaper | 1.39 | 1.68 |
| réglisse† | licorice | 1.73 | 1.07 |
| tulipe† | tulip | 2.17 | 1.42 |
| salle de bains | bathroom | 1.09 |  |
| ceinture† de sécurité | seat_belt | 1.44 |  |
| fougère† | fern | 1.7 | 0.28 |
| assiette† | dish | 1.15 | 0.69 |
| laine | yarn | 1.86 | 1.61 |
| cigarette† | cigarette | 1.31 | 1.98 |
| harpe | harp | 1.2 | 1.57 |
| crêpe† | pancake | 1 | 1.90 |
| **Feminine Incongruent** | | |  |
| vis | screw | -1.25 | 0.42 |
| pieuvre | octopus | -1.43 | 0.61 |
| carte | map | -0.63 | 0.91 |
| aile | wing | -1.01 | -0.14 |
| hache† | hatchet | -1.71 | -0.65 |
| poignée† de porte | doorhandle | -1.04 |  |
| sauterelle† | grasshopper | -1.28 | 0.42 |
| caverne | cave | -1.9 | -0.16 |
| voiture† de course | racecar | -0.98 |  |
| fourmi† | ant | -1.08 | -0.19 |
| bière† | beer | -1.47 | 1.63 |
| charrue† | plow | -1.54 | 0.35 |
| épée† | sword | -2.24 | 0.21 |
| arche† | ark | -2.09 | 0.18 |
| moustache† | mustache | -2.38 | 0.67 |
| basse† | bass_guitar | -2.56 | 1.06 |
| barbe | beard | -4.03 | 1.03 |
| **Masculine Congruent** | | |  |
| ballon† | ball | -1.02 | -0.62 |
| timbre | stamp | -1.21 | 0.43 |
| arc | arch | -1.06 | -0.26 |
| fer à cheval | horseshoe | -0.93 |  |
| gant† | glove | -1.17 | 0.02 |
| neurone | neuron | -1.24 |  |
| lion† | lion | -1.9 | -0.29 |
| drapeau† | flag | -1.57 | -0.72 |
| tuba† | tuba | -1.64 | 0.78 |
| bac à sable | sandbox | -0.64 |  |
| fusil | rifle | -1.71 | -0.73 |
| pied de biche | crowbars | -2.11 |  |
| boeuf | ox | -2.16 | 0.32 |
| **Masculine Incongruent** | | |  |
| foulard | scarf | 2.1 | 1.19 |
| biscuit† | cookie | 2.79 | 0.24 |
| plateau† | tray | 1.65 | 0.00 |
| linge | laundry | 1.81 | 0.95 |
| oreiller† | pillow | 1.64 | 1.31 |
| basilic | basil | 1.44 | 0.47 |
| pot† de fleurs | flowerpot | 0.94 |  |
| garde-manger | pantry | 1.73 |  |
| repas | meal | 1.14 | 0.25 |
| coquillage† | seashell | 1.42 | 0.42 |
| ongle | fingernail | 1.16 | 0.07 |
| papillon† | butterfly | 1.76 | 0.40 |
| réfrigérateur | refrigerator | 1.33 | 0.03 |
| lit† | bed | 0.95 | 0.42 |
| boa† | boa_constrictor | 1.1 | 0.24 |

†Regularity could be inferred from one or more of the following sources: Boloh & Ibernon, 2013; Desrochers & Brabant, 1995; Holmes & de la Bâtie, 1999; Holmes & Segui, 2004; Lyster, 2010; Seigneuric et al., 2007; Taft & Meunier, 1998
